# Intersection Vehicle Turning Control for Fully Autonomous Driving Scenarios

**DOI:** 10.3390/s21123995

**Published:** 2021-06-09

**Authors:** Zhizhong Ding, Chao Sun, Momiao Zhou, Zhengqiong Liu, Congzhong Wu

**Affiliations:** School of Computer and Information, Hefei University of Technology, Hefei 230009, China; zzding@hfut.edu.cn (Z.D.); sunchao7610@mail.hfut.edu.cn (C.S.); zqliu@hfut.edu.cn (Z.L.); wcz114773@hfut.edu.cn (C.W.)

**Keywords:** autonomous driving, vehicle turning control, VANET, model predictive control

## Abstract

Currently the research and development of autonomous driving vehicles (ADVs) mainly consider the situation whereby manual driving vehicles and ADVs run simultaneously on lanes. In order to acquire the information of the vehicle itself and the environment necessary for decision-making and controlling, the ADVs that are under development now are normally equipped with a lot of sensing units, for example, high precision global positioning systems, various types of radar, and video processing systems. Obviously, the current advanced driver assistance systems (ADAS) or ADVs still have some problems concerning high reliability of driving safety, as well as the vehicle’s cost and price. It is certain, however, that in the future there will be some roads, areas or cities where all the vehicles are ADVs, i.e., without any human driving vehicles in traffic. For such scenarios, the methods of environment sensing, traffic instruction indicating, and vehicle controlling should be different from that of the situation mentioned above if the reliability of driving safety and the production cost expectation is to be improved significantly. With the anticipation that a more sophisticated vehicle ad hoc network (VANET) should be an essential transportation infrastructure for future ADV scenarios, the problem of vehicle turning control based on vehicle to everything (V2X) communication at road intersections is studied. The turning control at intersections mainly deals with three basic issues, i.e., target lane selection, trajectory planning and calculation, and vehicle controlling and tracking. In this paper, control strategy, model and algorithms are proposed for the three basic problems. A model predictive control (MPC) paradigm is used as the vehicle upper layer controller. Simulation is conducted on the CarSim-Simulink platform with typical intersection scenes.

## 1. Introduction

Currently, the research and development of advanced driver assistance systems (ADAS) mainly focus on the oncoming market demand [1]. It is conducted by companies such as Tesla, Google, Baidu, etc., and the traffic situation they envision is usually that of humans driving vehicles, and with autonomous driving vehicles (ADV) or driverless vehicles occurring concurrently on the roads. In order to acquire the information of the vehicle itself and the environment, which is necessary for decision-making and controlling, the ADVs under development now are normally equipped with a lot of sensing systems, e.g., globe positioning system, lidar, millimeter wave radar, infrared radar, video system or vision system, and so on. The sensor information is processed by advanced or intelligent processing units. The current solutions of ADAS or ADV still have some problems [2]. Firstly, those equipped sensing systems might not absolutely guarantee the reliability of driving safety due to their performance degradation caused by bad weather, lack of light, obstacles, blind areas, etc. Secondly, it might take too long for the advanced or intelligent algorithms to extract needed information from the sensed signals such as video due to the computational complexity. For example, if we want a vehicle with a speed of 160 km/h to make decisions for autonomous driving in a time interval that the vehicle moves every 0.5 m distance, all the computations have to be finished in 22.5 milliseconds. Thirdly, those sensing systems, particularly lidar, increase the cost of vehicle production considerably.

It could be anticipated that in the future there are some roads, areas or cities where all the vehicles are totally ADVs, i.e., without any manual driving vehicles in the traffic. To improve the driving safety and to reduce the production cost, in such scenarios, the way of environment sensing, traffic instruction indicating and vehicle controlling might be different from what is used in current ADAS or ADV. We assume that a more sophisticated VANET (vehicle ad hoc network) should be an essential infrastructure for future ADV transportation since it can provide much more information with much more efficiency by V2X communications (i.e., vehicle-to-vehicle, vehicle-to-infrastructure, vehicle-to-pedestrian, etc.). This anticipation motivates the work of the article.

The statistical data of traffic accidents shows that many accidents are caused by the vehicle turning at road intersections [3]. Therefore, it is an important issue to control the vehicle behavior properly in such situations, moreover, controlling of vehicle turning at a road intersection is one of the most complicated problems to be solved in an ADV scenario. The problem mainly consists of three basic issues, i.e., target lane selection, trajectory planning and calculation, and vehicle controlling and tracking. In this paper, strategy, model and algorithms concerning intersection vehicle turning control of ADVs are proposed for the above three fundamental problems. The MPC (model predictive control) [4] paradigm is used as the vehicle upper layer controller. Simulation is conducted on the CarSim-Simulink platform with typical intersection scenes [5]. The main contributions of this paper are to:

1. Propose an approach to the problem of controlling the turning maneuver at intersections for ADV scenarios, which is based on V2X communication instead of various sensing systems, such as lidar, millimeter radar, and video system. It could be expected that the cost of cars could be reduced significantly with such a solution.
2. Propose a simple and feasible strategy for target lane selection considering the characteristics of fully ADV scenarios. Target lane selection is a relatively difficult problem in a non-fully ADV scenario.
3. Design and implement an MPC-based upper layer controller for vehicle self-driving and conduct extensive simulations by CarSim-Simulink cross platform.

The rest of this article is organized as follows: The research works related to ours are explored in Section 2. Section 3 discusses ADV scenarios and turning control. In Section 4, the simulation verification and data analysis of the proposed method are carried out. Finally, the conclusion and further research direction are given.

## 2. Related Work

The pioneer manufacture of ADV now develops cars that will drive together with human-driving vehicles on roads. They do not reveal the detail of their solutions, such as the trajectory calculation and vehicle control. However, it could be speculated that they prefer a general solution suitable to all road scenarios, rather than considering particular road shapes such as cross road or intersection [6]. This is due to the two factors. First, there is no easy way currently to acquire enough traffic instruction information and to detect road shape reliably before VANETs are deployed or highly detailed electronic maps are available. Secondly, random events or behaviors of human driving make it quite difficult to make control decisions that are effective in a whole procedure, for example, target lane selection when ADVs are turning.

In order to generate a correct vehicle turning which can prevent the risk of collision, the most important issue is the geographical trajectory planning and calculation for vehicles. There are a lot of literatures reporting the works related to turning trajectory, but they are mainly for transportation or road design [7,8,9,10]. For example, [7,8] use the chart analysis to show that the trajectories might be quite different for different drivers according to the actual traffic data. By analyzing and clustering the highly diversifying real turning tracks of human driving, [9] models the turning track by a Euler curve connected with a straight line at each end, to obtain continuously varying curvature. Later a five-segment turning trajectory is proposed, by inserting a circular arc between two Euler curves [10]. To form a driving trajectory from the geographical track, the relationship of velocity or acceleration versus time has to be established. The study [10] presents the mean velocity profiles for different vehicle types and road geometry by clustering method. A cubic function of speed change at turning intersection is proposed in [11], many constraint conditions determine each parameter in this cubic function, for example, residual coefficient, vehicle speed and acceleration, and unknown quantities. These unknown quantities reflect the behavioral differences caused by individual characteristics and inter attributes of drivers. These differences are modeled as random variables, and finally, parameters are determined by statistical method.

In order to solve the problem of multilane vehicle path conflict, [12] makes each turning vehicle choose a fixed lane by setting left turn guide lines. In [13], the lane selection behavior of traffic flow from branch road to trunk road at four continuous intersections of urban trunk road is measured. Lane selection behaviors are divided into two parts: temporary lane selection behavior and target lane selection behavior. Considering the different factors that influence the two parts above, such as drivers’ characteristics, lane attributes, the expected maximum utility of direct lane, etc., a joint probability model is constructed to select the lane with the highest probability for each vehicle.

The third issue is the controlling of vehicle. The popular structure of the controlling unit is hierarchical [14,15,16,17,18,19,20,21]. That is, the upper control layer is mainly to generate appropriate control variables, usually including acceleration and front-wheel angle. The lower control layer is a vehicle dynamics controller that converts the outputs of the upper layer to actuator input variables, such as throttle opening, steering wheel angle, etc. The work of this paper mainly focuses on the design of the upper controller, letting the lower control layer be implemented on a matured vehicle dynamic simulation platform such as CarSim.

The main target of the upper layer controller is to track its reference input. Currently, commonly used methods include classical proportional integral differential (PID) control [14,15], sliding mode control [16,17] and model predictive control (MPC) [4,18,19,20,21]. The paper [14] uses PID to realize the vehicle’s longitudinal upper control on the automatic highway. The study in [15] designs a forward control strategy and completes the formation coordination adaptive cruise system by improving the PID proportion coefficient. Nevertheless, PID upper layer controller normally considers only error feedback and has a relatively fixed structure. Therefore, the performance control effect is not satisfactory when the external conditions change. Hence, a scheme called sliding mode control has been proposed that does not require a fixed system structure and has the advantages of fast response and insensitive to disturbance. The work in [16] uses a cascade control system in which the inner loop uses the sliding mode control to ensure the trajectory tracking of the formation. In [17], a coupled sliding mode control method is proposed to improve the control performance and stability of the two-way platoon. However, this method needs to overcome the chattering when approaching the equilibrium point. The most recent works [4,18,19,20,21] are based on MPC, which is a class of optimization-based control paradigms with flexible structure and objective function. The study in [4] constructs a quadratic objective function to minimize the trajectory error, and the objective functions of [18] and [19] are based on fuel efficiency, and [20,21] proposes a predictive controller derived from the infinite norm with the aim to ensure that the distance between the front and rear vehicles is always greater than the minimum safe distance [20].

Several other works have introduced data-driven methods to predict turning vehicle trajectories and vehicle control. The host vehicle determines the turning trajectory by training the front vehicle trajectory in the data set [22,23] and relies on cameras, radar, GPS, and other sensors to finish vehicle control [24]. Generally speaking, this kind of vehicle turning control relying on a large number of sensors has high complexity, the accuracy of the perceived peripheral information cannot be guaranteed, and the cost is also significantly higher than that based on the V2X communication [25].

To the best of our knowledge, however, there is no literature focusing on the fully ADV scenarios where VANET could be expected to be an essential infrastructure of the future transportation system. Such an expectation motivates us to address the issue with different trains of thought and different solutions.

## 3. Turning Control for ADV Scenarios

As mentioned, the turning control at intersection mainly has three basic problems to solve, i.e., target lane selection, trajectory planning and calculation, and vehicle controlling and tracking, which are addressed in this section. It should be clarified first that the design or the approach proposed in this paper is based on the following considerations or assumptions:

- The computational load of algorithms is as low as possible so that they could run on inexpensive embedded systems, while maintaining real-time processing capability.
- By using a dedicated positioning system, instead of GPS or mentioned sensors, the future VANET could provide position information for all the vehicles as accurate as to tens of centimeters (actually, such a positioning device is under developed [26,27]).
- Information exchanged over or provided by VANET should be as less as possible since wireless channel capability would be a bottleneck of ADV application in crowded traffic cases.

### 3.1. Scenario Description and Problem Formulation

**Figure 1:**
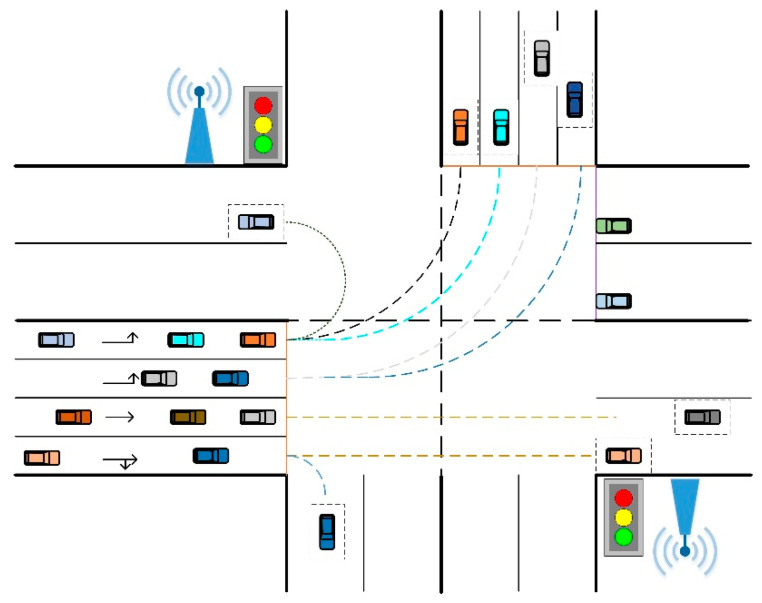
A typical multi-lane intersection scenario.

As shown in Figure 1, we consider a signalized intersection with multiple turning lanes and multiple target lanes. Road side units (RSUs) are distributed among both sides of the road, and all vehicles on the road are ADVs which are equipped with on-board units (OBUs). The communication delay and transmission contents loss can be not considered in the turning scene. For these conditions, this paper designs a turning control system that can achieve the following three functions.

(1) According to the driving requirement, the vehicles entering the intersection can realize the automatic control of turning left, turning right and turning around.
(2) When turning vehicles are released from multiple turning lanes of the signalized intersection, the turning control system needs to solve the problem of path conflict among turning vehicles to balance the exit traffic flow as much as possible, and to consider as few lane changes as possible after turning.
(3) In the process of turning, vehicles should shorten the distance from the vehicles in front as far as possible to improve the traffic efficiency of the intersection with the condition of ensuring a safe distance from the vehicles in front.

To realize vehicle control more effectively, vehicles approaching intersections can receive essential messages from RSUs. Basic motion states and some additional data of each vehicle can be also transferred to each other by the OBUs.

In this paper, RSUs are only required to send messages to OBUs without receiving back from OBUs, which will significantly reduce the workload of RSUs. The format and content of messages from RSUs and OBUs are shown in Table 1.

In an RSU message, the number of starting lanes, target lanes and the coordinates of stop line of each road will be broadcast. The state can be either yes or no to indicate whether access in this direction. The remaining time (RT) is the remaining time till the state changes. The combination of direction, state and remaining time can indicate the remaining time of green light in each direction.

In an OBU message, vehicle ID, location, and velocity are the basic information of vehicles. The status indicates the behavior that the host vehicle is expected to take, whose value can be −1, 0, or 1, representing decelerating to stop, keeping running in current motion states, or accelerating till the desired velocity, respectively. Moment tells when to change the behavior, which is related to status. It stands for the decelerating moment when the status equals −1, and for the accelerating moment when the statues equals 1.

### 3.2. Driving Control Frame of Turning Vehicle

**Figure 2:**
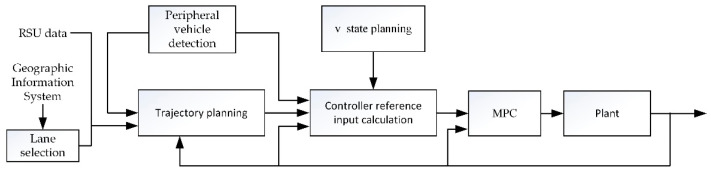
System structure of turning vehicle for ADV.

The turning control frame proposed in this paper is shown in Figure 2. It is mainly composed of five modules: target lane selection, driving trajectory planning, controller reference input calculation, MPC controller, and plant. In order to solve the problem of vehicles trajectory conflict in multiple turning lanes, this paper first designs a target lane selection algorithm that generates the coordinates of the exit point for each vehicle.

The trajectory planning module establishes the geographical movement track from the turning starting point to the endpoint. Then the reference input controller calculation module generates the geographical motion trajectory with a timestamp, to form the reference trajectory. In this paper, a hierarchical design is adopted for path tracking. The upper layer controller uses the MPC module to generate control variables, acceleration, and front-wheel direction. The lower layer controller completes vehicle dynamics control, relying on the dynamic simulation platform CarSim mainly, which is the plant module in the block diagram. The peripheral vehicle detection module in our system needs just the information of position, speed, and heading values of other vehicles to detect whether the surrounding environment is abnormal in the process of the vehicle turning, which could be acquired through the vehicle to vehicle (V2V) communications, but the needed ego vehicle state information such as pose, can be provided by the local OBU. The v-state planning module broadcasts the state of the car that mainly refers to when the car in front changes its state. For example, status = 1 and $Moment=t_{Ad}$ indicate that the car in front starts to accelerate at time $t_{Ad}$, which is convenient for the car behind to plan the trajectory.

### 3.3. Target Lane Selection

As mentioned above, the purpose of target lane selection is to solve the multilane turning vehicle path conflict, ensure traffic efficiency, and make the vehicles change lanes as few as possible after turning. Therefore, our lane selection algorithm is applied before vehicles enter the intersection, and it mainly depends on the direction of the motion of the vehicles at the next intersection and the lane selection of vehicles ahead.

The specific design taking left turn as an example is as follows: assume that there are *M* left turning lanes and *N* target lanes at the current intersection (these data are obtained by RSU broadcast at the intersection). The core idea of the lane selection scheme is to transform the *M*-to-*N* lane selection problem into a 1-to-*N′* lane selection problem, and the vehicles in each left source lane do not affect each other, so the scheme needs to receive the road selection results of the front vehicle in the current left turn lane only. In addition, few vehicles turn left at the next intersection when turning left at the current intersection, so the allocation of the target lane should be inclined from the second lane. The specific steps are as follows:

Step 1: The target lane is divided into *M* parts averagely, and the average number of lanes corresponding to each source lane is *k* = *N/M.*

Step 2: Since it is not exactly evenly divided, the remaining margin of the target lane is *T* = *N* − k*M. 

Step 3: Each vehicle builds a queue of length *M* in its own OBU. Firstly, the initial value of each queue is *k*, that is [k,k,k⋯k], where the number of elements is M. Then, the margin of the target lane is allocated from the second lane. Finally, the following queue can be obtained in all OBUs, that is:

(1) $$\left[\underset{M}{\underbrace{k,\underset{T}{\underbrace{k+1,k+1,\cdots k+1}},k\cdots k}}\right]$$

Step 4: Each OBU converts the queue to the target lane required by the current host vehicle.

Step 5: Call the lane selection function of 1-to-*N′*, and choose the empty lane close to its target lane each time.

As for the lane selection function of 1-to-*N′*, it is relatively simple since it is not involving the problem of path conflict. The vehicles are firstly grouped according to *N′*, and the rear vehicle selects the lane that the front vehicle does not select but that is closest to the desired lane. For example, if the vehicle will turn right at the next intersection, it expects the lane closest to the right naturally. If the ahead vehicles in the same group did not select the rightmost lane, the current turning vehicle will select that lane.

### 3.4. Trajectory Planning and Calculation

Driving track planning only considers the geographical track that the vehicle should follow. On this track, when and where the vehicle starts and stops, the speed of the vehicle for each position is analyzed and controlled as separate problems. Here the ideal track of a turning vehicle can be assumed to be an arc combined with a straight line. Its rationality mainly lies in two points: (I) the driver’s turning track is close to the arc in most turning scenes; and (II) the steering wheel angle is basically fixed in actual turning (that is, the front wheel angle is basically the same). According to Ackerman’s steering geometry idea [28], we have R=L/δ,where *L* denotes the longitudinal distance in meters between the center of the front and rear wheels, δ denotes the front wheel deflection in radians, and *R* denotes the turning radius in meters. If the speed is basically fixed, its corresponding track is a certain arc. However, if the terrain is too limited to follow the arc, or if the vehicle detects a danger to the surrounding vehicles while making the turn, we need to replan the trajectory (see Figure 2). Here this paper focuses on revealing a scenario in which a turn can be completed in an arc combined with a straight line.

Corresponding to our algorithm, OBU receives four key point coordinates broadcasted by RSUs, including the location coordinates of the starting road stop point *X_s_*(*x*_0_,*y*_0_), the starting road extension line one point *X*_s1_(*x*_1_,*y*_1_), the ending road stop point *X_f_*(*x_N_*_0_,*y_N_*_0_), and one point of the ending road extension line *X_f1_*(*x_N_*_1_,*y_N_*_1_). Here, the extended line point is not arbitrarily selected, but preselected by RSU. It can represent the yaw angle of the road combined with the stop line point. The coordinates here are GPS positions. Taking *X_s_* as an example, *x*_0_ is the longitude of the position while *y*_0_ is the latitude, and others are similar. The outputs are the position of starting road stop *X_s_*, ending road stop *X_f_*, arc start *A* arc end *B* the center of a circle *T* and corner radius *R* start road yaw angle $\varphi_{s}$, terminal road yaw angle $\varphi_{f}$. The yaw angle here refers to the angle between the vehicle’s main body direction and the north pole direction. Moreover, it should be noted that point A and point *X_s_*, point B and point *X_f_* may coincide. The specific algorithm implementation is shown as follows.

Step 1: A straight line is determined according to two points. Thus, the yaw angle of the starting road is determined from the stop point of the starting road and the extension line of the road. The yaw angle of the terminal road is determined from the stop point of the terminal road and the extension line of the road.

Step 2: Calculate the linear equation of the starting road and the ending road. They are $L_{1}$,$L_{2}$.

Step 3: Calculate the linear equation of the stop line of the start road and the end road.

Step 4: Calculate the intersection coordinate $M(x_{M},y_{M})$ of two roads. Save the intersection point if there is an intersection. Otherwise, the two roads are parallel.

Step 5: The distance between the center of the circle and the two roads is equal, and the determined circle must be inscribed to the lane line, so the unique circle is determined. Thus, the center of the circle and the radius of the arc can be obtained.

(2) $$\left(T,R)=f(L_{1},L_{2}\right)$$

Step 6: Since the arc track is determined, the starting point and ending point of the arc in the whole turning process are obtained.

The above algorithm is applicable to left turn, right turn, and U-turn. When the difference between $\varphi_{s}$ and $\varphi_{f}$ is π or −π, it means a U-turn. Similarly, if the difference is between 0 and π, or between −2π and −π, it means turning left; otherwise, it means turning right.

The driving track planning above has planned out geographical movement track, turning speed limit and the acceleration of turning vehicle at the current intersection should be considered next to make the geographical path time stamped. Therefore, this paper will introduce the controller reference input calculation algorithm from the above two aspects respectively as follows.

#### 3.4.1. Maximum Speed of Turning Vehicle

The maximum speed limit of turning vehicles mainly depends on the terrain and the performance of vehicles. The speed limit that most vehicles can reach will be adopted here. At this speed, there will be no sideslip during the turning process of vehicles. Corresponding to our algorithm, it is the minimum of the following two values: (I) the speed limit for turning vehicles at the current intersection, and (II) literature [28,29] give the speed limit under steady-state steering characteristics by using a vehicle model with two degrees of freedom [28,29]. The relation between speed and turning radius is formulated as follows.

(3) $$R=\frac{L}{\delta }(1+Kv^{2})$$

Transformed from Formula (3), the relation between the speed and turning radius can be obtained:

(4) $$v=\sqrt{\frac{R*\delta /L-1}{K}}$$

where *K* is called the stability factor of a vehicle, and it is defined as the follows.

(5) $$K=\frac{m}{L^{2}}(\frac{l_{f}}{k_{2}}-\frac{l_{r}}{k_{1}})$$

where *k*_1_, *k*_2_ denote the cornering stiffness of the front and rear wheels; $l_{f}$ and $l_{r}$ are the distance from the center of mass to the center of the front and rear wheels, *L* is the wheelbase length of the vehicle, and *m* is its mass. Suppose all the parameters of the vehicle can be obtained from its electronic units and the turning radius can be obtained from the RSU, Equation (4) will determine one speed limit *v_h_* for the turning vehicle. On the other hand, there might be a turning speed limitation *v_l_* given also by the intersection RSU, therefore an actual maximal value could be obtained by selecting a minimal one, that is:

(6) $$v_{cmax}=\min(v_{l},v_{h})$$

The calculated result is basically consistent with the actual driving turning speed.

#### 3.4.2. Acceleration Model of Turning Vehicle

Generally speaking, when entering the intersection, the initial speed *v*_0_ is not more than the maximum turning speed *v_cmax_*. In this way, our problem is transformed into the speed change from *v_0_* to *v_cmax_*. This paper uses the idea of clustering seen in Reference [9], which clusters the average acceleration of vehicles at multiple traffic intersections, and then fits the relationship between the average acceleration value *a* and the road turning radius *R* to construct the function.

(7) a=g(R)

At this time, using the calculated mean acceleration to replace the acceleration change of the whole intersection can not only simplify the vehicle control process, but also be easier to achieve in the era of electric vehicles.

Thus, we can get the speed change process of the whole intersection.

(8) $$v(t)=v_{0}+at$$

When the vehicle speed reaches *v_cmax_*, the vehicle passes through the intersection at a constant velocity *v_cmax_*.

### 3.5. Vehicle Controlling and Tracking

Since the vehicle does not follow our reference trajectory without error at the time, the application of MPC in this paper is mainly to assist the upper dynamic adjustment control of the vehicle.

**Figure 3:**
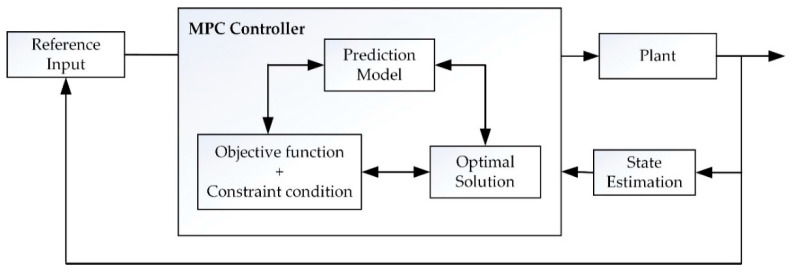
MPC control principle.

The control principle of MPC is shown in Figure 3. Firstly, the real-time state value and the expected state value of the controlled object (notice that the expected trajectory is discretized to get known) are taken as the input of the MPC controller. Then, the prediction module in the MPC controller calculates the state values of the future *N_p_* time points according to the state update equation as Formula (9). Finally, the optimization module of the MPC controller establishes the loss function according to the minimum error value between the predicted state value and the expected state value and solves the control input value applied to the controlled object.

Here, the state values this paper selects include lateral position error, longitudinal position error and yaw angle error and their derivative, which is expressed by $\bar{\xi }={\left(\dot{\bar{y}},\dot{\bar{x}},\bar{\psi },\dot{\bar{\psi }},\bar{Y},\bar{X}\right)}^{T}$, the outputted control variables are the current front wheel angle and acceleration, expressed as u(δ,a). Referring to the vehicle dynamics equation [30,31,32], the following state transfer equation is obtained:

(9a) $$\bar{\xi }(k+1)=A\bar{\xi }(k)+Bu(k)+D$$

(9b) $$A=\left[\begin{array}{cccccc} 1 & T & 0 & 0 & 0 & 0\\ 0 & 1-\frac{T*(2*C_{f}+2*C_{r})}{m*v_{x}} & \frac{T*(2*C_{f}+2*C_{r})}{m} & \frac{-T*(2*C_{f}*l_{f}-2*C_{r}*l_{r})}{m*v_{x}} & 0 & 0\\ 0 & 0 & 1 & T & 0 & 0\\ 0 & \frac{-T*(2*C_{f}*l_{f}-2*C_{r}*l_{r})}{I_{z}*v_{x}} & \frac{-T*(2*C_{f}*l_{f}-2*C_{r}*l_{r})}{I_{z}} & 1-\frac{T*(2*C_{f}*l_{f}^{2}+2*C_{r}*l_{r}^{2})}{I_{z}*v_{x}} & 0 & 0\\ 0 & 0 & 0 & 0 & 1 & T\\ 0 & 0 & 0 & 0 & 0 & 1 \end{array}\right]\;D=\left[\begin{array}{c} 0\\ -v_{x}*T-\frac{T*(2*C_{f}*l_{f}-2*C_{r}*l)}{m*v_{x}}\\ 0\\ \frac{-T*(2*C_{f}*l_{f}-2*C_{r}*l_{r})}{I_{z}*v_{x}}\\ 0\\ T \end{array}\right];\;\;\;\;\;\;B=\left[\begin{array}{cc} 0 & 0\\ \frac{2*C_{f}*T}{m} & 0\\ 0 & 0\\ \frac{2*T*C_{f}*l_{f}}{I_{z}} & 0\\ 0 & 0\\ 0 & -T \end{array}\right].$$

The cost function is constructed according to the principle of minimum error.

(10) $$\begin{array}{l} \underset{\Delta u(k)}{\min}\;\;\;J(k)=\sum_{i=1}^{N_{p}}{\left\|\bar{\xi }(k+i|k)\right\|}_{Q}^{2}+\sum_{i=1}^{N_{c}}{\left\|\Delta u(k+i|t)\right\|}_{R}^{2}+\rho \varepsilon^{2}\\ \;s.t.\;\;\;\;u_{\min}(k+j)<u(k+j)<u_{\max}(k+j)\\ \;\;\;\;\;\;\;\;\Delta u_{\min}(k+j)<\Delta u(k+j)<\Delta u_{\max}(k+j) \end{array}$$

where *j* = 0,1,2,… *Nc*−1 and *i*|*k* stands for the *i*th prediction step at time step *k. N_p_* represents the prediction horizon length. *N_c_* represents the control horizon length. The parameters Q, R, and ρ ∊ [0, 1] are chosen in order to have a good trade-off among reference trajectory, gap policy tracking and actuators excitation. The parameter ε is the relaxation factor, which makes the optimization function solvable.

After solving Equation (10) in each control cycle, a series of control input increments in the control time domain are obtained:

(11) $$\Delta U_{t}^{*}={\left[\Delta u_{t}^{*},\Delta u_{t+1}^{*},\Delta u_{t+2}^{*},\cdots ,\Delta u_{t+N_{c}-1}^{*}\right]}^{T}$$

The first element in the control sequence is acted on the system as the actual control input increment, that is:

(12) $$u(t)=u(t-1)+\Delta u_{t}^{*}$$

After entering the next control cycle, it repeats the above process, so as to realize the trajectory tracking control of the vehicle.

## 4. Simulink and Experimental Results

In this section, we make some simulation experiments to test our algorithm as well as the MPC controller on CarSim-Simulink. The quality of communication is assumed ideal, the traffic scenes are simulated to test lane selection algorithm and vehicle turning control with right-hand traffic rules such as China, the United States, etc.

### 4.1. Target Lane Selection Simulation

**Figure 4:**
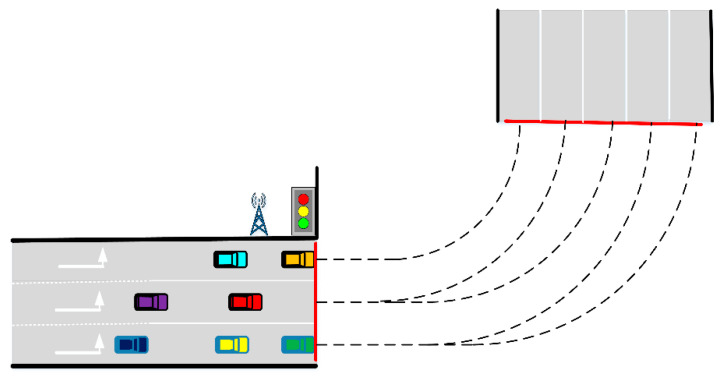
The initial scene of the multi-lane simulation.

As shown in Figure 4, there are seven vehicles from three different lanes in the same direction. The width of each lane is set to be 3.5 m, and three left lanes and five target lanes are to be selected. Taking the first car in the first lane as an example, the Y-axis coordinate of the vehicle traveling on the left-side through lane is set 0, the X-axis coordinate is also set 0. More vehicle fundamental parameters and initial conditions are shown in Table 2 and Table 3 respectively. The performance parameters of the vehicle selected in CarSim are shown in Table 3, where *L*, *l_f_*, *l_r_*, *k*_1_ and *k*_2_ are the same as the meaning in formula (5) and $I_{z}$ denotes the moment of inertia about the Z-axis.

**Figure 5:**
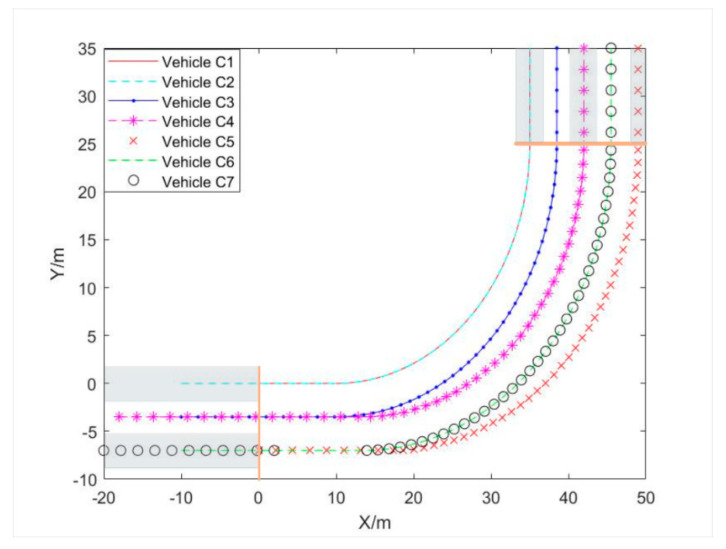
Track coordinates of all seven vehicles.

The simulation results are shown in Figure 5. All vehicles in the leftmost lane choose target lane 1, red vehicle C3 and purple vehicle C4 in the second left turn lane choose target lane 2 and 3 respectively, green vehicle C5 in the third left turn lane chooses target lane 5, yellow vehicle C6 and blue vehicle C7 in the third left turn lane choose target lane 4. According to the previous target lane selection strategy, the 3- to 5-lane selection scheme should correspond to the following: the first left turn lane corresponds to target lane 1 only, the second left turn lane corresponds to target lane 2 and 3, and the third left turn lane corresponds to target lane 4 and 5. The results in Figure 5 correspond to the expected behavior of vehicles precisely.

### 4.2. Turning Maneuver Simulation

According to the results of target lane selection above, the *M*-to-*N* lane selection problem can be transformed into a 1-to-*N′* lane selection problem, and the vehicles in different lanes do not affect each other, therefore, this paper further verifies the turning maneuver control of two vehicles in the same lane. The speed of the front vehicle is *v*_1_, the target lane is *roadSelect1*, the speed of the rear vehicle is *v*_2_, and the target lane is *roadSelect2*. Notice, *roadSelect1* can be the same as or different from *roadSelect2*. The performance metrics chosen to compare the control configurations are the error of tracking reference trajectory and the safe distance. 

The control configurations analyzed in the comparisons are three turning scenarios. The following turning scenarios are considered.

1. L–TC: left turn control scenario. The driving behaviors of both the front and rear vehicles are to turn left, and the rear vehicle needs to make a strategic judgment on whether it can pass the intersection at the current moment and control the safe distance. If it can pass the current intersection, and its speed is less than the maximum speed of the left turn scenario, it accelerates to the maximum turning speed with fixed acceleration, and then drives at a constant speed. Considering the control of the waiting area included in the left turn control scenario here, this paper distinguishes it by the flag bit of strategy judgment, that is flagLeftTurn (one has to note that there is red light control for the left turn by default.). If flagLeftTurn = 1, it means that vehicles can turn left through the current intersection, and if flagLeftTurn = 2, it means that the vehicle stops in the waiting area, otherwise, it means that it cannot pass.

2. R-TC: right turn control scenario. The driving behaviors of both the front and rear vehicles are turning right. Here we need to consider whether there is red light control scenario, and this paper uses the flag bit flagRightTurn to distinguish. Generally, there is no waiting area for the right turn, so the flag bit flagRightTurn = 1 indicates that the vehicle can turn right through the current intersection. On the contrary, it means that it cannot pass.

3. U-TC: U-turn control scenario. It is similar to the right turn scenario. Considering whether there is red light control, *flagUTurn* = 1 indicates that the vehicle can turn around and pass the current intersection, otherwise it cannot pass.

Furthermore, in order to more accurately reflect the performance of the algorithm designed in this paper, we also need to consider the performance of the control algorithm in different road scenarios, such as different front and rear vehicle speeds, different distances between two vehicles when the front and rear vehicles just enter the intersection, and different turning radii of the road. Here, this paper intercepts several typical intersection road scenarios of Hefei City as virtual experimental roads. Through field measurement, the turning radius at the intersection of Danxia Road and Bainiao road is 11 m, that at the intersection of Jinzhai South Road and Ziyun road is 25 m, and that at the intersection of Fanhua Avenue and Jinzhai south road is 35 m. The selection of distance and speed of the front and rear vehicles are shown in Table 4 below. Additionally, it is worth noting that the rear vehicle here is the host vehicle.

#### 4.2.1. Verify the Effectiveness

**Figure 6:**
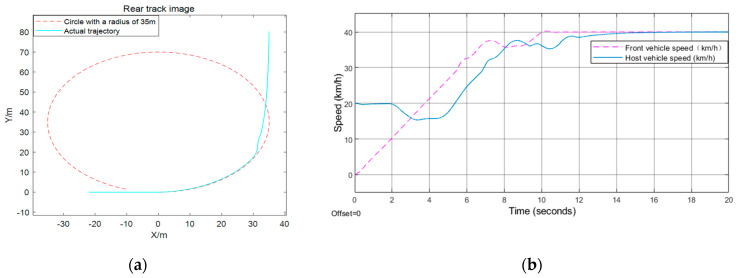
Turn left test result: (**a**) rear track image; (**b**) front and rear vehicle speed.

**Figure 7:**
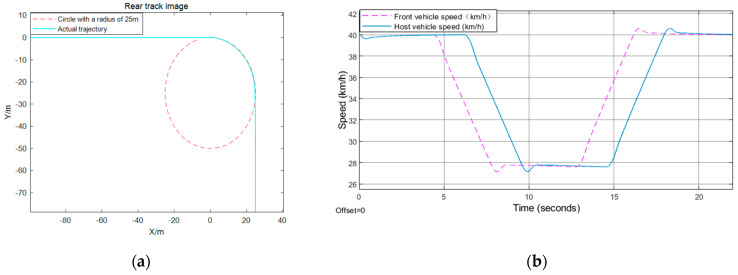
Turn right test result: (**a**) rear track image; (**b**) front and rear vehicle speed.

**Figure 8:**
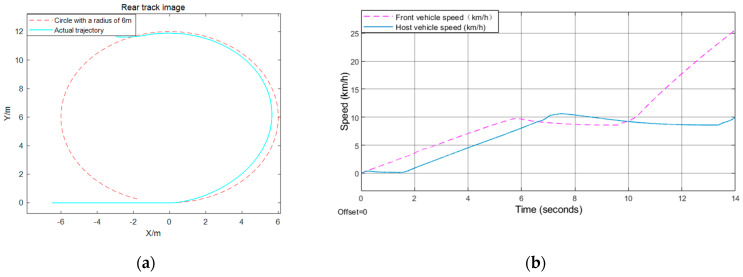
U-turn test result: (**a**) rear track image; (**b**) front and rear vehicle speed.

The verification of effectiveness for the proposed model is mainly conducted under CarSim17, which is a widely used platform to simulate vehicle motion. This paper uses CarSim17 and Simulink for CO-Simulation, and uses XY_Graph, scope, matlabFunction to process the simulation data. The specific results are shown in Figure 6, Figure 7 and Figure 8. Here, this paper selects the position, speed of the car behind, and the distance from the front vehicle under the track as the observation measures to test the effectiveness of the algorithm.

The front vehicle in Figure 6 starts from the stop line, the distance between the rear car and the front car is 17 m, and the speed of the rear vehicle is 20 km/h. However, in Figure 7, the initial speed of the front and rear vehicle speed is 40 km/h, the distance between the two is 15 m, the distance between the front vehicle and the stop line is 80 m. Nevertheless, the front vehicle starts from the stop line just right, and the rear vehicle is also still, with a distance of 1.5 m from the front vehicle in Figure 8.

Figure 6 shows that the behavior of the car behind is to keep a constant speed for a period of time, then decelerate until a state where the distance from the vehicle in front is equal to the fixed safety time distance, and the speed is the same as that of the car in front, then keep a constant speed for *t*_h_ seconds at the current velocity, and then accelerate to the maximum speed limit. According to formulas (4) and (7), when the turning radius is 6 m, the acceleration is 0.5 *m*/*s*^2^, and the maximum turning speed is 10.0 km/h. When the turning radius is 25 m, the acceleration is 1 *m*/*s*^2^ and the maximum turning speed is 28.0 km/h, and when the radius is 35 m, the acceleration is 1.25 *m*/*s*^2^ and the maximum turning speed is 36.2 km/h. In the behavior depicted in Figure 7, it firstly maintains the current speed for 8 s, then decelerates evenly for 1.3 s, whose acceleration is −1 *m*/*s*^2^, which means that it decelerates until the stop line is just equal to *v_cmax_*, and then passes the intersection at a constant speed. Because the initial distance between the vehicles ahead and behind is greater than the fixed time interval, the front vehicle begins to decelerate after sending the deceleration time stamp to the rear car. At this time, the rear car calculates its behavior according to the current distance. As for the behavior depicted in Figure 8, since the static safety distance is fixed at the beginning, the rear car first stops for *t_h_* seconds, then accelerates to the maximum speed limit, while the car in the front accelerates directly to the maximum speed limit.

#### 4.2.2. Test the Performance of the Proposed Method

**Figure 9:**
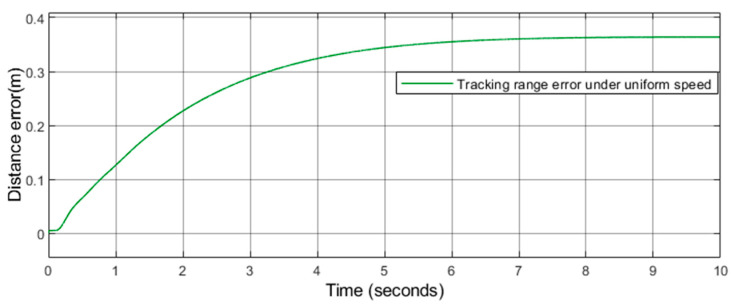
Tracking range error under uniform speed.

**Figure 10:**
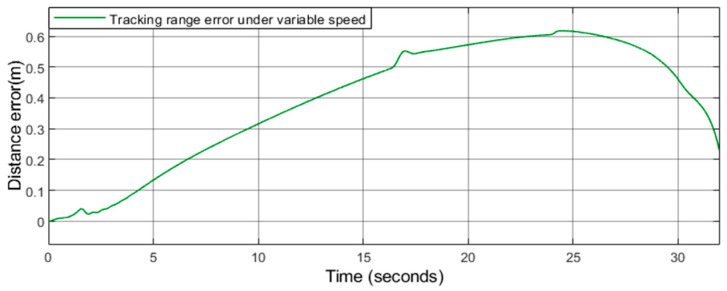
Tracking range error under variable speed.

As shown in Figure 6, Figure 7 and Figure 8, further analysis of the error between the actual track and the reference track can be made. This paper takes the rear car as the object of explanation and uses the scope function to draw the error between the actual track and the reference track at each moment. As shown in Figure 9 and Figure 10, we can see that the maximum error at driving with uniform speed is 0.35 m, and the maximum error at driving with variable speed is 0.61 m. This paper maintains the consistency of the system parameters including turning left, turning right, and U-turn.

**Figure 11:**
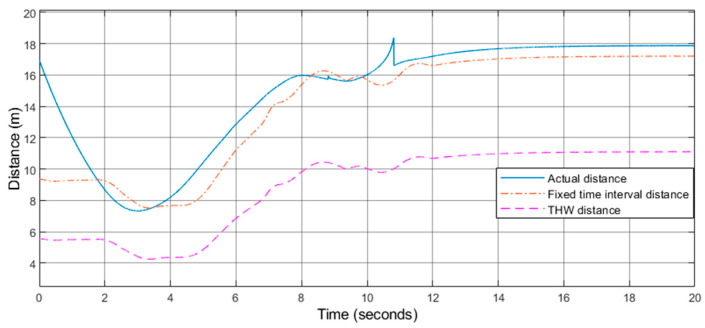
Distance image of front and rear vehicles.

Furthermore, this paper analyzes the safety of the algorithm. Taking the left turn as an example shown in Figure 11, the distance between the host car and the front car is described, where the blue solid line refers to the actual distance. The orange dash dot line represents the fixed time interval distance, and the red dotted line represents the THW distance which is defined by the minimum safety distance of forwarding collision, which is *d*_min_ = *v*_rel_ * *T*_thw,_ here *T*_thw_ is 1.2 s [33]. The actual distance curve is always higher than the minimum distance curve. In a word, the security of the algorithm can be guaranteed. Moreover, the distance between the front and rear vehicles controlled by the control algorithm is close to the fixed time interval distance, which is the distance control strategy commonly adopted by the formation now [14].

## 5. Conclusions

This paper studies a practical turning vehicle control algorithm based on the internet of vehicles. According to the information obtained by V2V and V2I communication, including the surrounding vehicles, road information, etc., this article designs the turn lane selection scheme, and establishes the trajectory planning and upper MPC vehicle control model. Finally, in order to verify the reliability and efficiency of the algorithm, this paper uses the simulation platform CarSim to carry out the simulation test. The experimental results show that the algorithm has good reliability and robustness.

There is still more interesting work to be further studied. As this paper experiments only on our simulation platform, we will further consider the real vehicle test and improve the control performance. Through the actual vehicle data, the corresponding parameters of the algorithm will be further debugged.

## Figures and Tables

**Table 1:** Messages defined for RSU and OBU.

RSU Message	OBU Message
Number of lanes	Vehicle ID
Location of the Stop line	Location
Direction	Velocity
State	Status
Remaining Time	Moment

**Table 2:** Vehicle starting positions in the multilane simulation.

Vehicle No.	1	2	3	4	5	6	7
X(m)	0	−10	−8	−18	0	−10	−20
Y(m)	0	0	−3.5	−3.5	−7	−7	−7

**Table 3:** Vehicle dynamic parameters.

Vehicle Mass	Body Size	*L*	$l_{f}$	$l_{r}$	$k_{1}$	$k_{2}$	$I_{z}$
1723 kg	5 m	2.6 m	1.232 m	1.468 m	66,900 N/rad	42,700 N/rad	4175 kg·m²

**Table 4:** Front and host vehicle speed and distance between host vehicle and front vehicle.

$v_{pre}$	$v_{ego}$	$d_{1}$	$d_{2}$	$v_{l}\;$	*R*	Direction
0 km/h	20 km/h	0 m	17 m	40 km/h	35 m	Left
40 km/h	40 km/h	80 m	15 m	40 km/h	25 m	Right
0 km/h	0 km/h	0 m	1.5 m	30 km/h	6 m	U-turn

where $v_{pre}$ denotes the speed of front vehicle, $v_{ego}$ denotes the host vehicle speed, $d_{1}$ represents the distance from the front vehicle to the stop line, $d_{2}$ represents distance from host vehicle to front vehicle, and *R* is the road radius. $v_{l}$ denotes the max velocity of the vehicle on the road.

## Data Availability

Not applicable.
